# Chloroplast genome of a pair of *Triticum aestivum* L. recombinant inbred lines with significant difference in seed size

**DOI:** 10.1080/23802359.2021.1934155

**Published:** 2021-06-14

**Authors:** Li Huaizhu, Hanjun Xue

**Affiliations:** a School of Chemistry and Chemical Engineering, Xianyang Normal University, Xianyang City, Shaanxi, China; b Xianyang Academy of Agricultural Sciences, Xianyang City, Shaanxi, China

**Keywords:** *T. aestivum*, chloroplast genome

## Abstract

Wheat (*Triticum aestivum L.*), one of the most important crops belong to the *Triticum* genus of family Poaceae. Some of important cytoplasmic genes come from chloroplast genome. In this study, the chloroplast genome of a pair of *T. aestivum* recombinant inbred lines were sequenced, assembled, and annotated. Our results show this chloroplast genome consists of 137 unique genes, including 87 protein-coding genes, 42 tRNA genes, and 8 rRNA genes. A maximum-likelihood phylogenetic tree based on 15 chloroplast genomes revealed that the two *T. aestivum* are closely related to *Triticum* genus. The chloroplast genome could be used for wheat species identification, cytoplasmic inheritance gene functional study and breeding.

Wheat (*Triticum aestivum* L.) from *Triticum* genus of family Poaceae is one of the most important crops in the world. The improvement of yield has always been a critically important and a main aim for wheat breeding over the past years (Wang et al. 2015). As an important part of wheat yield, grain size is largely determined by nuclear coding genes. However, recent research has suggested that non-nuclear genes may affect the seed size as well (Du et al. 2016). Chloroplast genome contributes to a large part of non-nuclear genes. The type species wheat chloroplast genome has been sequenced in 2017 (Zimin et al. 2017). To date (1/20/2021), there are only two varieties of *T. aestivum* chloroplast genome in GenBank (Accession no. CM022232, NC_002762). In this study, we reported two chloroplast genomes from a pair of *T. aestivum* recombinant inbred lines which obvious differences in grain size, based on Illumina Hiseq pair-end sequencing data.

Samples were collected from wheat germplasm resources garden of Xianyang Normal University (Geographic coordinates: 34°22′49.18″N, 108°44′6.99″E; Altitude: 418 m), frozen and preserved at a refrigerator. The specimen was deposited in the herbarium of Xianyang Normal University (http://www.xysfxy.cn/, Huaizhu Li, lihuaizhu121@126.com) under the voucher no. Xsy200511001 and Xsy200511002. Total genomic DNA was extracted with modified CTAB method and preserved in refrigerator (Stefanova et al. 2013). Genome sequencing was performed by Illumina NovaSeq 6000 at Biomarker Technologies Corporation (Illumina, San Diego, CA). Low-quality sequences were filtered with Q30 (base Phred quality score of ≥30). Total high quality reads were mapped to reference (Triticum aestivum cultivar Chinese Spring chloroplast, complete sequence, CM022232) using Bowtie2 (Langmead and Salzberg 2012) and the mapped reads were extracted. All extracted reads were assembled by SOAPdenovo2-src-240 (Luo et al. 2012) and generate several contigs. These contigs were aligned to the reference sequence (*Triticum aestivum* cultivar Chinese Spring chloroplast, complete sequence, CM022232) by BLAST + v2.11.0 (Camacho et al. 2009) to determines site and direction. There is only one inverted repeat region (IRa) in these contigs. All of these contigs and the second inverted repeat region copy (IRb, inverted IRa) were constructs a majority consensus sequence by CAP3-linux-x86-64 (Xiaoqiu Huang and Madan 1999). The assembled chloroplast genome were annotated and manually corrected using Geneious v8.0.2 (Kearse et al. 2012), and were deposited into GenBank (Accession nos. MW548259 and MW575926) .

The chloroplast genome of the two *T. aestivum* were 135,884 bp and 135,886 bp, respectively. It possessed a typical quadripartite structure with two identical copies of inverted repeat regions separated by large and small single copy region. The large single copy region (LSC) in the two *T. aestivum* were 79,988 bp and 79,990 bp, respectively. The small single copy region (SSC) was 12,790 bp, and the inverted repeat regions (IRs) were 21,553 bp. A total of 137 genes were annotated, including 87 protein-coding genes, 42 tRNA genes, and 8 rRNA genes. GC content of the complete chloroplast genome is 37.8%. The chloroplast genomes structure, gene order of the two *T. aestivum* were nearly identical. There are only four SNPs (including 2 deletion/insertion) in the LSC region between them.

Molecular phylogenetic tree was constructed to elucidate the evolutionary relationship of the two *T. aestivum* with other species in Poaceae. A total of 15 chloroplast genome (every genome only include one inverted repeat region), including the two *T. aestivum* and 13 other Poaceae species were multiple aligned by MAFFT v7.468 (Kazutaka Katoh et al. 2002). Subsequently the maximum likelihood phylogenetic tree was generated by RAxML v7.2.8 (Stamatakis 2006) with 1000 bootstrap replicates (Figure 1). Phylogenetic analysis showed that the two *T. aestivum* were closely related to the *Triticum* genus (Figure 1). Evolutionary relationship is consistent with the morphological classification. The complete chloroplast genome of the two *T. aestivum* would lay foundations for *Triticum* species identification, cytoplasmic inheritance gene functional study and wheat breeding.

**Figure 1.**
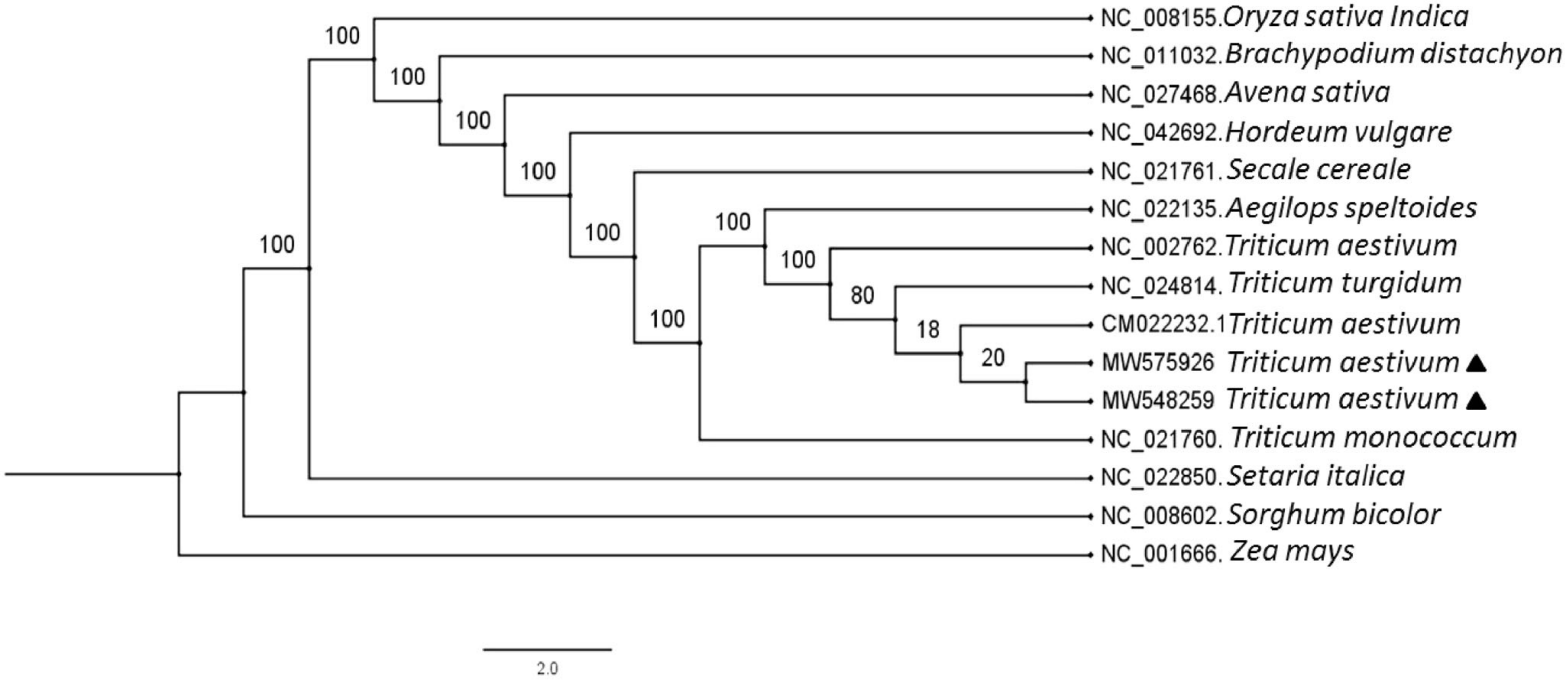
Maximum-likelihood phylogenetic tree base on 15 chloroplast genomes. The accession numbers are shown in the figure. Bootstrap support values based on 1000 replicates are displayed on each node. *Zea mays* as outgroup. Labeled by black triangle are *T. aestivum* in this study.

## Data Availability

The genome sequence data that support the findings of this study are openly available in GenBank of NCBI at (https://www.ncbi.nlm.nih.gov/) under the Accession no. MW548259 and MW575926. The associated BioProject and Bio-Sample numbers are PRJNA702909, SAMN17982933 and SAMN17982934, respectively.
